# The Influence of Inflammation on Fibrinogen Turnover and Redistribution of the Hemostatic Balance to a Prothrombotic State in High On-Treatment Platelet Reactivity-Dual Poor Responder (HTPR-DPR) Patients

**DOI:** 10.1155/2019/3767128

**Published:** 2019-07-17

**Authors:** Grzegorz Biolik, Dariusz Gajniak, Maciej Kubicz, Damian Ziaja, Krzysztof Ziaja, Wacław Kuczmik

**Affiliations:** ^1^Department of General, Vascular Surgery, Angiology and Phlebology Faculty of Katowice Medical University of Silesia, Faculty of Medicine in Katowice, Medical University of Silesia, Poniatowskiego Str 15, 40-055 Katowice, Poland; ^2^Department of Anaesthesiology and Intensive Care, Upper-Silesian Medical Centre of the Silesian Medical University, Ziołowa 45, 40-635 Katowice, Poland; ^3^Physiotherapy Unit, Department of Physiotherapy, Faculty of Health Sciences, Medical University of Silesia, Poniatowskiego Str 15, 40-055 Katowice, Poland

## Abstract

Knowledge about the influence of inflammation on platelet function and relocation of hemostatic balance to hypercoagulable state is still unclear. We compared two groups of patients who suffer from acute vs. chronic inflammatory process and additionally present high on-treatment platelet reactivity-dual platelet resistance. We did not found any differences in platelet aggregation between both investigated groups, but patients who suffer from chronic inflammation presented stronger relocation of the hemostatic balance to the hypercoagulability. A high concentration of prothrombin fragment F1+2 together with higher activity of von Willebrand factor in critical limb ischemia shows more exaggerated fibrinogen turnover although the blood concentration of this factor was in normal range. We concluded that high on-treatment platelet reactivity-dual platelet resistance and intensified inflammation are linked with elevated platelet and fibrinogen turnover to counteract proper hemostatic balance in favor of a prothrombotic state.

## 1. Introduction

Platelets play a key role in the pathophysiology of coronary, cerebral, and peripheral artery diseases, and platelets are especially a key in arterial thrombosis [1–9]. The significance and impact of acute and chronic inflammatory processes on platelet function, clot formation, and atherogenesis are still unclear; however, these processes seem to play a pivotal role in the reallocation of hemostatic balance to a hypercoagulable state [1, 10–15]. Knowledge about the influence of inflammation on platelet function, not only is regarded to thrombus formation, may result in the development of more clinically effective treatment strategies and a better understanding of HTPR-DPR.

## 2. Aim

The aim of our study was to show the influence of weak vs. strong inflammatory reactions on the movement of hemostatic balance towards a hypercoagulable state due to increased fibrinogen turnover in patients who have normal concentrations of this clotting factor in their blood. The changes in hemostatic balance were assessed in two groups of patients: an AMI group (acute myocardial infarction) and a CLI group (critical limb ischemia).

## 3. Material and Methods

This study was performed on two groups of patients who suffered from HTPR-DPR: 23 CLI patients who qualified for tight amputation due to advanced necrotic changes (Rutherford class 6). There were also 26 AMI patients who qualified for the study; the AMI patients were included 3-5 days after percutaneous coronary intervention (PCI), drug-eluting stent (DES) implantation to the coronary arteries, and additionally suffered from low ejection fraction (EF) <45%.

Common inclusion criteria were as follows: all investigated patients received dual antiplatelet therapy (DAPT), 75 mg of clopidogrel and 75 mg of aspirin daily. Contrary to the CLI group, all AMI patients received a loading dose of clopidogrel (600 mg) and aspirin (300 mg) just before the endovascular procedure on the day of admission to the hospital. All patients suffered from HTPR-DPR. HTPR-DPR was defined as the area under the curve (AUC) from Multiplate^®^ impedance aggregometry: HTPR-DPR was defined as AUC > 436 for the ADP test and >300 AUC for the ASPI test. All patients presented with a normal blood fibrinogen concentration (e.g., 1.8–3.5 g/L) and elevated blood concentration of C-reactive protein (e.g., >5 mg/L). The detailed characteristics of both investigated groups are presented in Table 1.

The exclusion criteria were as follows: diabetes, arrhythmia, chronic kidney diseases (CKD) in stage 3B or higher, or receiving additional oral anticoagulation.

All of the study participants provided written consent to take part in the study after reading the study protocol.

The study was conducted after obtaining permission from the Local Bioethical Commission within a broader research project concerning platelet hyperreactivity in patients with critical limb ischemia.

### 3.1. Blood Sampling

Venous blood was collected into citrate vacutainer tubes (3.2% sodium citrate) for ROTEM^®^ analysis and into hirudin vacutainer tubes (15.4 *μ*g/mL, final concentration of hirudin) for Multiplate^®^ investigations. Obtained samples were evaluated within 15 min, and the reagents for both methods were used following the manufacturer's recommendations.

### 3.2. Thromboelastometry

Rotational thromboelastometry was performed with a ROTEM^®^ Delta (TEM International, Munich, Germany). Two tests were performed, EXTEM and FIBTEM, according to the standard protocols supplied by the manufacturer. The following parameters were analyzed: clotting time (CT), clot formation time (CFT), amplitude after 10 min of measurement (A10), amplitude after 20 min of measurement (A20), and maximum clot firmness (MCF) for EXTEM and A10, A20 and MCF for FIBTEM. Calibration of the device was performed by representatives of the manufacturers. Quality control was performed every day before measurements. The “hypercoagulable profile” was recognized when the value of MCF was above the upper limit of the norm for MCF (e.g., supranormal value of MCF >70 mm for EXTEM or >25 mm for FIBTEM using definitions described by Görlinger et al.) [10].

### 3.3. Aggregometry

Impedance aggregometry measurements were performed on a Multiplate^®^ function analyzer (Dynabate Information System, Munich, Germany; with software version V2.03.11) using the standard reagents and protocols recommended by the manufacturer for the four tests: ADP, ASPI, COL, and TRAP. Aggregation was measured as the area under the curve (AUC/mm^2^). As mentioned previously, HTPR-DPR was diagnosed when the AUC was >436 for the ADP test and >300 for the ASPI test. Similar to thromboelastometry, the calibration was performed by representatives of the manufacturer, and quality control was performed every day before measurement.

### 3.4. Statistical Analysis

All data were presented as the mean ± standard deviation for normally distributed variables and as medians and percentages for nonnormally distributed variables. For further comparisons between both groups, the following tests were used: Wilcoxon, Mann-Whitney *U*, and other nonparametric alternatives for the analysis of variance, and Kruskal-Wallis. Statistica 9 software (StatSoft Inc., Tulsa, USA) was used for the statistical data analysis.

## 4. Results

### 4.1. Aggregometry

In terms of blood platelet aggregation, the ADP, ASPI, COL, and TRAP tests did not reveal any statistically significant differences between the investigated groups. The results are presented in Table 2.

### 4.2. Thromboelastometry

EXTEM test analysis showed that all patients in the CLI group presented “supranormal” values on the MCF test (hypercoagulability), while the AMI group results were between “normal” and “supranormal” for the same test. On the other hand, FIBTEM test analysis showed unequivocally “supranormal” values on the MCF test (hypercoagulability) in the CLI group and “normal” (9-25 mm) values in the AMI group. The results are presented in Table 3.

### 4.3. Laboratory Tests

The fibrinogen concentration was within normal values in both groups, and there was no statistically significant difference between groups.

There was no statistically significant difference in mean platelet volume (MPV) between both groups. Interestingly, the mean value of MPV was above 9 fL in both groups. This finding may be associated with an increased fraction of immature platelets.

In terms of inflammatory process assessment, the values of hs-CRP were significantly higher in the CLI group than in the AMI group.

The von Willebrand factor (vWF) activity levels were significantly higher in the CLI group, but there was no difference in vWF antigen concentration between the groups. The results demonstrated not only a higher intensity of inflammatory processes in patients qualified for amputation but also a shift in hemostatic balance towards hypercoagulable state.

There was also a statistically significant difference in the concentration of prothrombin fragment 1+2 (F1+2) between groups, with higher values in the CLI population. Importantly, the mean concentration was above the upper limit of the normal range in both groups. This observation suggests that the process of increased fibrinogen turnover is present in both groups but is more exaggerated in the CLI group.

## 5. Discussion

Clinically, elevated hs-CRP levels and HTPR have been characterized as important risk factors for adverse ischemic cardiovascular events, although the data are still controversial [1, 11–20]. In many papers, both are presented as independent risk factors for myocardial infarction (MI), periprocedural myocardial infarction (PMI), or stent thrombosis. C-reactive protein (CRP) plays an important role in the processes of thrombogenesis and thromboembolism [1, 21–25]. Together with other proinflammatory cytokines, it plays an important role in the production of clotting factors that contribute to an enhanced prothrombotic state [4, 25–27]. These disturbances in the hemostatic system are mainly mediated by the increased production of fibrinogen and von Willebrand factor as well as platelet activation; in cases of intensified inflammation, there is observed increased turnover fibrinogen, von Willebrand factor, and platelets [15, 26, 28–31]. Therefore, the blood concentration of some of these factors may be in a normal range and falsely suggest a normocoagulable state.

Routine laboratory assays for prothrombin time and activated partial thromboplastin time are unable to detect a hypercoagulable state because they do not entirely reflect overall hemostatic balance [32, 33].

Two methods, rotational thromboelastometry ROTEM^®^ and Multiplate^®^ aggregometry, are useful for recognizing a hypercoagulable state related to participation of platelets, fibrinogen, and factor XIII [17, 31–34].

ROTEM^®^ provides further information about thrombus generation and total clot strength that closely correlates with plasma thrombogram assays, the gold standard test for thrombin generation. In this method, fibrin-based clot strength is dependent mainly on fibrinogen and factor XIII concentrations, while the platelet component of clot strength is defined as the difference in shear modulus measured with and without platelet inhibition (MCE_platelet_ = MCE_EXTEM_‐MCE_FIBTEM_) [17, 29, 31–36]. This suggests that platelet contribution in overall clot strength in ROTEM^®^ is related to binding and tightening of the fibrin fibers. The main limitation of this method is the lack of permanent inhibition of platelet activity in the FIBTEM test, even in healthy subjects. This limitation was described by Michos and many other investigators [19]. The last observations show that, in HTPR patients, the platelet aggregation ability is many times stronger, and aggregation is still observed (as the residual) if cytochalasin D and IIb/IIIa inhibitors are used [31–35, 37–40].

Analysis of the ROTEM^®^ amplitude (A) and maximum clot firmness (MCF) values for the EXTEM and FIBTEM tests carried out by Görlinger et al. provided the scientific basis for the division of the treated patients into three groups, subnormal, normal, and supranormal, according to the reference ranges for these tests [17].

The ROTEM^®^ supranormal values for amplitude (A) and maximum clot firmness (MCF) suggest a hypercoagulable state but do not offer simple reason for this state [17].

We analyzed two groups of patients with HTPR-DPR. There were no statistically significant differences in the intensification of HTPR-DPR (platelet aggregation), PLT count, fibrinogen concentration or, most importantly, tissue factor, and thrombomodulin concentrations. In both investigated groups, we observed intensified von Willebrand factor activity and an increased prothrombin factor F1+2 concentration that was more intensified in the CLI group. Because there is a strong and linear correlation between the concentration of this protein (F1+2) and the intensification of fibrinogen degradation, it seems that the differences observed in EXTEM and FIBTEM were not related to platelets but were related to the other clotting factors, mainly to fibrinogen degradation and turnover.

It seems that the ROTEM^®^ may be a promising method for recognizing high risk patients who suffer from HTPR-DPR because comparison of HTPR-DPR, factor F1+2 concentration, and the results of EXTEM/FIBTEM could be helpful for proper recognition of the inflammatory impact on hemostatic balance and for estimation of the real risk of thrombosis. It may be helpful for DAPT modification (such as for a drug's dosage) with or without additional anticoagulation to achieve tailored (personalized) therapy without increasing the risk of bleeding. However, as was shown by P. Gurbel and his team, there are not any methods that can simultaneously measure and analyze the relationships between inflammation (demonstrated by different elevated markers), platelet reactivity, and hypercoagulability [1, 10, 17]. Therefore, further research for strategies specifically targeting high-risk patients seems to be necessary and would be useful for personalized therapeutic management.

## 6. Conclusion

HTPR-DPR and intensified inflammation are linked with elevated platelet and fibrinogen turnover to counteract proper hemostatic balance in favor of a prothrombotic state.

## 7. Study Limitations

This study was a pilot investigation. The relatively small number of investigated patients in both groups was the reason for a very restrictive inclusion and exclusion criteria; notably, we had to eliminate cardiac patients who suffered from acute bacterial or viral infections.

In the paper, we did not analyze the D-dimer concentration between the two groups. The D-dimer concentration was very high in the CLI group due to thrombosis in ischemic/necrotic limbs; therefore, the groups were not comparable.

## Figures and Tables

**Table 1:** Characteristics of the study group.

	CLI	AMI	*P* value
Age (yrs)	66 ± 4.2	63 ± 7.6	NS
Male sex, *n* (%)	21 (91.3)	19 (88.4)	NS
BMI (kg/m^2^)	21.1 ± 1.9	22.3 ± 1.1	NS
INR (*r.v. 0.8-1.2*)	1.0 ± 0.05	0.9 ± 0.07	NS
Index APTT (*r.v. 0.84-1.16*)	0.93 ± 0.1	0.98 ± 0.7	NS
Fibrinogen (g/L) (*r.v. 1.5-4.5*)	3.0 ± 0.5	2.8 ± 0.5	NS
Platelets (×10^9^/L) (*r.v. 150-450*)	283.2 ± 91.3	219.8 ± 44.1	NS
Platelet distribution width (%) (*r.v. 8.0-18.0*)	13.6 ± 3.2	17.2 ± 2.1	NS
Mean platelet volume (fL) (*r.v. 8.0-12.0*)	9.3 ± 1.6	9.3 ± 0.7	NS
White blood cells (×10^9^/L) (*r.v. 4.5-10.0*)	10.2 ± 3.0	9.4 ± 1.9	NS
Red blood cells (×10^12^/L) (*r.v. 4.5-5.5*)	4.4 ± 0.5	4.6 ± 0.2	NS
Hemoglobin (g/dL) (*r.v. 13.0-18.0*)	14.37 ± 1.4	14.0 ± 1.8	NS
Hematocrit (%) (*r.v. 40-54*)	42.6 ± 4.9	41.3 ± 5.4	NS
hs-CRP (mg/L) (*r.v. <5.0*)	60.8 ± 12.9	6.4 ± 3.1	<0.006
Thrombomodulin (*μ*g/L) (*r.v. 1.8-4.4*)	2.36 ± 0.74	2.63 ± 0.78	NS
von Willebrand antigen (U/mL) (*r.v. 0.51-7.71*)	5.02 ± 1.35	4.41 ± 1.15	NS
von Willebrand activity (%) (*r.v. 55-140*)	65.6 ± 16.4	36.7 ± 11.1	<0.01
Prothrombin fragment F1+2 (nmol/L) (*r.v. 0.2-2.78*)	4.02 ± 2.2	2.28 ± 1.7	<0.01
Tissue factor (*μ*g/L) (*r.v. 28-255*)	123.1 ± 26.0	107.8 ± 31.4	NS

Abbreviations: CLI: critical limb ischemia; AMI: acute myocardial infarction; BMI: body mass index; hs-CRP: high-sensitivity C-reactive protein.

**Table 2:** Results of aggregometric findings.

MEA (AUC)			
ADP (mm^2^) (*r.v. 534-1220*)	866.8 ± 113.8	769.8 ± 138.5	NS
ASPI (mm^2^) (*r.v. 745-1361*)	485.3 ± 167.1	412.6 ± 98.7	NS
COL (mm^2^) (*r.v. 459-1166*)	722.7 ± 196.2	625.8 ± 151.8	NS
TRAP (mm^2^) (*r.v. 941-1563*)	1494.9 ± 226.1	1364.9 ± 440.0	NS

Abbreviations: ADP/ASPI/COL or TRAP: standard test; AUC: area under the curve (mm^2^); r.v.: referential value.

**Table 3:** Results of thromboelastometric findings.

EXTEM			
	CLI	AMI	*P* value
CT (s) (*r.v. 38-79*)	52.5 ± 8.0	50.6 ± 5.4	NS
CFT (s) (*r.v. 34-159*)	50.3 ± 17.4	71.4 ± 15.5	<0.04
A10 (mm) (*r.v. 43-65*)	70.0 ± 7.7	63.0 ± 4.4	<0.01
A20 (mm) (*r.v. 50-71*)	73.5 ± 6.5	68.2 ± 3.8	<0.02
MCF (mm) (*r.v. 50-72*)	73.9 ± 6.3	69.0 ± 3.5	<0.03
INTEM			
CT (s) (*r.v. 100-240*)	184 ± 7.3	247.5 ± 11.2	NS
CFT (s) (*r.v. 30-110*)	54.5 ± 7.75	79 ± 10.9	NS
A10 (mm) (*r.v. 44-66*)	62 ± 9.6	60.5 ± 9.35	NS
A20 (mm) (*r.v. 50-71*)	67 ± 9.93	66 ± 9.15	NS
MCF (mm) (*r.v. 50-72*)	67 ± 9.81	66.5 ± 9.25	NS
FIBTEM			
A10 (mm) (*r.v. 7-23*)	32.1 ± 13.6	20.5 ± 5.7	<0.01
A20 (mm) (*r.v. 8-24*)	33.3 ± 14.0	21.7 ± 5.9	<0.01
MCF (mm) (*r.v. 9-25*)	33.6 ± 14.1	21.9 ± 6.1	<0.02

Abbreviations: CT: clotting time; CFT: clot formation time; A10: amplitude after 10 min of measurement; A20: amplitude after 20 min of measurement; MCF: maximum clot firmness; r.v.: referential value of CT, CFT, A10, A20, and MCF for specific test. EXTEM, INTEM, and FIBTEM are standard tests.

## Data Availability

The database and the results are available for reviewers.
